# Pediatricians' attitudes, experience and referral patterns regarding complementary/alternative medicine: a national survey

**DOI:** 10.1186/1472-6882-7-18

**Published:** 2007-06-04

**Authors:** Anju Sawni, Ronald Thomas

**Affiliations:** 1 Department of Adolescent Medicine, Children's Hospital of Michigan, Wayne State University School of Medicine, Detroit, MI 48201-2119, USA; 2 Children's Research Center of Michigan, Children's Hospital of Michigan, Wayne State University School of MedicineDetroit, MI, 4820-2119, USA

## Abstract

**Background:**

To assess pediatricians' attitudes toward & practice of Complementary/Alternative Medicine (CAM) including their knowledge, experience, & referral patterns for CAM therapies.

**Methods:**

An anonymous, self-report, 27-item questionnaire was mailed nationally to fellows of the American Academy of Pediatrics in July 2004.

648 of 3500 pediatricians' surveyed responded (18%).

**Results:**

The median age ranged from 46–59 yrs; 52% female, 81% Caucasian, 71% generalists, & 85% trained in the US. Over 96% of pediatricians' responding believed their patients were using CAM. Discussions of CAM use were initiated by the family (70%) & only 37% of pediatricians asked about CAM use as part of routine medical history. Majority (84%) said more CME courses should be offered on CAM and 71% said they would consider referring patients to CAM practitioners. Medical conditions referred for CAM included; chronic problems (headaches, pain management, asthma, backaches) (86%), diseases with no known cure (55.5%) or failure of conventional therapies (56%), behavioral problems (49%), & psychiatric disorders (47%). American born, US medical school graduates, general pediatricians, & pediatricians who ask/talk about CAM were most likely to believe their patients used CAM (P < 0.01).

**Conclusion:**

Pediatricians' have a positive attitude towards CAM. Majority believe that their patients are using CAM, that asking about CAM should be part of routine medical history, would consider referring to a CAM practitioner and want more education on CAM.

## Background

There is growing interest among patients, physicians, healthcare administrators, and third party payers in complementary/alternative medicine (CAM). CAM refers to a large range of therapies outside the domain of mainstream Western medicine that are used for the purpose of medical intervention, health promotion, or disease prevention. The National Center for Complementary and Alternative Medicine (NCCAM) at the National Institutes of Health defines CAM as "a group of diverse medical and health care systems, practices, and products that are not presently considered to be part of conventional medicine."[1] Some examples of alternative therapies are: acupuncture/acupressure, botanical medicine, herbs, homeopathy, chiropractic, massage therapy, osteopathic manipulation, mega-vitamin therapy, nutritional supplements, mind-body medicine (hypnosis, biofeedback, meditation, yoga, Tai Chi, etc.), energy healing (Reiki, therapeutic touch), naturopathy, Ayruvedic medicine, traditional Chinese medicine, environmental therapies (magnets, lights), prayer and other healing spiritual practices.

The use of CAM in adults in the US has increased in the past several years from 34% to 42%[2]. The 2002 National Health Interview Survey (NHIS) of 31,000 adults reported that 49% of adults had used some form of CAM, excluding prayer for healing [3]. CAM use among children is also prevalent, particularly among children with chronic diseases such as cystic fibrosis, cancer, asthma, etc. with CAM use ranging from 11–80%. [4-15] Studies of CAM use in primary care pediatrics show a prevalence of 12–21%. [16-19] As a result of this interest and use of CAM among adults and children, primary care physicians are confronted with patients who want information about CAM or are using CAM.

CAM also appears to be gaining acceptance among conventionally trained physicians. Several studies assessing attitudes, beliefs and use of CAM by primary care physicians in the US, Canada and Europe report that 10–80% of physicians expressed an interest in CAM, want more education on CAM, have a positive attitude towards CAM, and consider referring patients for CAM [20][21-25] There are scant data on pediatricians' attitudes, beliefs and use of CAM in the US. To date there is one regional study done in 1997 that specifically looked at pediatricians' attitudes and experience with CAM in Michigan [26]. This study found that 83.5% of pediatricians surveyed believed some of their patients are using CAM but the majority believe this use constituted less than 10%. The majority of the pediatricians wanted more education about CAM and would consider referring for CAM. Another study by Kemper et al in 2001 assessed attitudes and recommendations of pediatricians regarding CAM for common pediatric conditions and found that 87% had been asked about CAM by their patients but did not feel comfortable discussing or recommending CAM therapies and wanted education on CAM [27]. The purpose of this study was to do a national survey to assess US pediatricians' attitudes, beliefs, experience, knowledge, referral patterns regarding CAM therapies and desire for continuing medical education (CME) regarding CAM therapies with the goal of helping to guide future research and medical education in CAM for pediatricians, both in training and practice.

## Methods

An anonymous, self-report, 27-item survey was mailed nationally to a random sample of US fellows of the American Academy of Pediatricians in July 2004. Pediatricians in training were excluded. A total of 3500 surveys were mailed and 648 (18.5%) were returned after one mailing. Data were analyzed for the 648 respondents.

The survey was developed by the authors and based on a survey used in a previously described study [26]. The survey consisted of 27 questions divided into demographic information, pediatricians' perception of their patients' use of CAM, whether their patients discussed their use of CAM with them, whether they asked about CAM use, personal and family use of CAM, whether they practiced any CAM therapies, their knowledge of CAM therapies and attitudes regarding the safety, harmfulness, effectiveness and their willingness to refer for 17 complementary/alternative therapies. A table format was used that listed the 17 CAM therapies; acupuncture/acupressure, aromatherapy, ayurvedic medicine, biofeedback, chiropractic, energy healing/polarity, herbs (North American, Chinese), high dose anti-oxidant vitamins, minerals, homeopathy, guided imagery, lifestyle diets (Ornish, marcrobiotics etc) massage therapy, prayer healing, reflexology, relaxation (yoga, mediation etc) touch. For each therapy the respondents indicated therapies practiced, personal/family use, referral for treatment, and rated the therapies as effective, safe, harmful or didn't know.

An informational letter was mailed with the survey instrument explaining what CAM therapies are and the reason for participating in the study. The study was approved by the Institutional Review Board of Wayne State University School of Medicine.

Statistical analysis employing SPSS software (SPSS version 11.5 Inc, Chicago, IL) provided descriptive summary statistics. Frequency distributions were generated for all variables. The Fisher-exact chi-square tests were used for bivarate comparisons, and significance was achieved at p < .05.

## Results

A total of 3500 surveys were mailed, 648 were returned after one mailing (18.5%) and data was analyzed for the 648 surveys returned. The results of the 648 surveys are reported. The pediatricians who responded have characteristics that are representative of the overall AAP membership. The median age ranged from 46–59 yrs, 52% were female, 81% Caucasian, 71% were generalists and 54% were in private practice. The majority went to medical school in the US (85%) and 56% have been practicing medicine for at least 15 yrs. (Table 1)

**Table 1:** Demographics of respondents

**Characteristics**	**Percentage (N = 648)**
**Gender**	
Female	52%
Male	48%
	
**Age**	
<35 yrs	18%
35–45 yrs	33%
46–59 yrs	35%
>59 yrs	14%
	
**Ethnicity**	
Caucasian	81%
Asian	9%
African-American	4%
Hispanic	3%
Others	2.5%
Native American	0.5%
	
**Where Born**	
U.S.	85%
Foreign	15%
	
**Where Attended Medical School**	
U.S.	85%
Outside U.S.	15%
	
**Years in Practice**	
< 15 yrs	55%
>15 yrs	45%
	
**Type of Practice**	
Private Practice	54%
Academic/Hospital-Based	30%
Other/HMO/PPO/Clinic/Public Health/ER	16%
	
**Pediatric Generalist**	71%
**Pediatric Sub-Specialist**	29%

### Perceptions of CAM use by pediatricians

Of the pediatricians surveyed, 96% believe their patients are using some form of CAM although the majority (72%) believe this constituted less than 30% of their patients; 80% stated that their patients ask them about CAM. The majority (76%) said they talk to their patients about CAM but most often (70%) the discussion is initiated by the family; only 37% ask about CAM use as part of routine medical history, although 79% said CAM use should be part of a routine pediatric medical history. Less than half (30%) reported using CAM therapies on their patients but only 18% had any formal training and most of the training was self-taught or CME (55%). The majority (84%) of the respondents want more CME courses on CAM, report that CAM modalities should be taught in medical school (80%), would consider referring patients to CAM practitioners(71%), would consider CAM therapies for themselves (75%) and almost half (49%) report personal use of CAM therapies (past or present). (Table 2)

**Table 2:** Pediatricians' perceptions and attitudes about CAM (N = 648)

	**Percentage**
Personal use of CAM	49%
Consider referring patients for CAM	74%
Talk to patients about CAM	75%
Parents initiate discussions about CAM	70%
Ask about CAM as part of routine medical history	37%
Patients ask pediatrician about CAM	80%

Table 3 lists particular medical problems for which pediatricians refer or consider referring their patients for CAM therapies. Medical problems most often referred or considered for referral were: chronic problems (86%) such as (headaches, abdominal pain, asthma, pain management), when conventional medical therapies failed (56%), diseases with no known cure (55.5%), behavioral problems (ADHD, nightmares) (49%), and psychiatric disorders (depression, anxiety, eating disorders) (47%). Fewer than 30% would refer for neurological disorders (seizures, cerebral palsy), cancer, autoimmune disorders (SLE, arthritis) and HIV.

**Table 3:** Medical problems for which pediatricians refer/consider referring for CAM therapies (N = 519)

	**Medical Problems**	**Percentage**
1	Chronic Problems (headaches, abdominal pain, asthma, pain management	86%
2	When conventional medical therapies failed	56%
3	Other chronic diseases with no known cure	55.5%
4	Behavioral problems (ADHD)	49%
5	Psychiatric Disorder (depression, anxiety, eating disorders)	47%
6	Neurological Disorders (seizures, muscular dystrophy, cerebral palsy)	28%
7	Cancer	26%
8	Autoimmune Disorders (SLE, arthritis)	26%
9	HIV	20.5%
10	Other	10%

### Attitudes towards specific therapies

Table 4 looked at usage (personal and practice), referral patterns, effectiveness, safety, and harmfulness for various CAM therapies among pediatricians responding.

**Table 4:** Use of CAM (personal and practice), referral patterns, safety/harmfulness, and effectiveness of various complementary/alternative therapies (N = 648)

**Therapies**	**Therapies practiced (%)**	**Self or Family use (%)**	**Refer for (%)**	**May be effective (%)**	**Safe (%)**	**May be harmful (%)**
Acupuncture/Acupressure	3	20.5	34	83	65	6
Aromatherapy	2.5	20	5	26	49	4
Ayurvedic	0.6	3	3	11	8	4
Biofeedback	5	11	52.5	79.5	66	1
Chiropractic	0.9	16	25	54	21	57
Energy healing/polarity Magnetic healing	0.4	6	4	16	24	9
Herbs (N American or Chinese)	5	17	10	52	15	61
High dose anti-oxidant vitamins/minerals	5	17	4	31	13	57
Homeopathy	2	7	5	32	24	23
Hypnosis	3.5	9	29	71	49	5
Imagery	6	10	21	51.5	46	2
Life style diet (Ornish, macrobiotic etc)	5	11	11	39	20.5	30
Massage therapy	8	34	39	75	66	3
Prayer for healing	9	18	9	49	54	8
Reflexology	1	8	5	21	27	3
Relaxation (yoga, meditation)	9	29	32	75	71	2
Touch Therapy	2	5	8	38	43	2

This table summarizes pediatricians' attitudes and practices about 17 common CAM therapies. Less than 10% of CAM therapies were practiced and of these the most common ones were: prayer for healing (9%), relaxation (yoga, mediation) (9%), massage therapies (8%), imagery (6%), herbs, megavitamins, lifestyle diet and biofeedback (5%). Therapies most commonly used in their personal life (themselves or family used) were: massage therapies (34%), relaxation (yoga, mediation) (29%), acupuncture/acupressure and aromatherapy (20.5%), prayer for healing (18%), herbs and megavitamins (17%), and chiropractic manipulation (16%). Therapies believed to be most effective were: acupuncture/acupressure (83%), biofeedback (79.5%), massage, relaxation (yoga, mediation) (75%), hypnosis (71%), chiropractic manipulation (54%), herbs (52%), imagery (51.5%) and prayer for healing (49%). Therapies believed to be safe were similar to those believed to be effective i.e. acupuncture/acupressure, biofeedback, massage, relaxation (yoga, mediation), prayer for healing, aromatherapy and hypnosis.

Therapies referred for were: biofeedback (52.5%), massage (39%), acupuncture/acupressure (34%), relaxation (yoga, meditation) (32%), hypnosis (29%) and chiropractic manipulation (25%). Therapies believed to be most harmful were: herbs (61%), megavitamins (57%), chiropractic manipulation (57%) and homeopathy (23%).

### Characteristics and factors related to positive attitudes towards CAM

Pediatricians who believe their patients are using CAM are in private, academic or hospital based practice (p < 0.01), generalists (p < 0.001), born in the US and went to US medical schools (p < 0.01), have patients who ask them about CAM (p < 0.001), talk to patients about CAM (p < 0.001), believe CAM should be part of the routine medical history (p < 0.01), and want more CME courses on CAM (p < 0.01). Pediatricians who talk to patients about CAM are generalists rather than a subspecialists and in private practice (p < 0.01). More female pediatricians report using CAM therapies personally (p < 0.02), would refer for CAM (p < 0.05) want more CME courses on CAM, and believe CAM should be part of the medical school curriculum (p < 0.05). Pediatricians who would refer for CAM are 45 yrs of age or younger, female, use CAM personally and talk to patients about CAM (p < 0.01).

## Discussion and conclusion

This national survey of pediatricians in the USA found that an overwhelming majority of pediatricians (96%) believe their patients are using some form of CAM and have been asked about CAM therapies by their patients (80%). Comparing this study to a similar study done by the authors in 1997 with Michigan pediatricians [26] indicates that seven years later more pediatricians believe their patients are using CAM (83.5% vs 96%), a larger percentage of their patients are using CAM (10% vs 30%) and more pediatricians are being asked about CAM (54% vs. 80%).

Even though the majority of pediatricians (70%) talk about CAM with their patients, the discussion is mostly initiated by the family. Less than half (37%) ask about CAM use as part of routine medical history. This finding is similar to that in the study by Kemper et al [27] who found that the majority (87%) of the pediatricians surveyed stated they had been asked about CAM but less than a quarter ask about CAM use as part of the medical history. Pediatricians' apprehension, lack of knowledge and training about CAM might be the reason why they do not routinely ask about CAM use. The fact that over three quarters of the pediatricians are interested in CME courses on CAM and believe that CAM modalities should be taught in medical school indicates that pediatricians are more acutely aware of their lack of knowledge and training in CAM and recognize the need for more education. Kemper et al [27] also reported that over 80% of the pediatricians' surveyed desired more education on CAM. Currently in US medical schools, there is no consistent or formal education and training in CAM. A Study done by Wetzel et al [28] in 1998 of 117 medical schools in the US found that, 64% of the medical schools stated they offered either an elective in CAM or the topic of alternative medicine was included into a required course, but there was tremendous diversity in the content, format and requirements among these courses in CAM.

Studies of other primary care physicians' attitudes towards CAM also indicate that the majority of primary care physicians believe their patients are using CAM, have a positive attitude towards CAM and want more education on CAM. [20][21-25] This increasing awareness, interest and communication about CAM among pediatricians may be reflective of the reaction to increasing trends in the use of CAM by adults [2,3]and children, [4-19] growing acceptance of CAM among conventionally trained pediatricians [26,27] and an increase in the amount of information on this subject (CME courses, medical journals, research, and the media, TV and internet).

Characteristics of pediatricians who believe their patients are using CAM are: generalists rather than sub-specialists, in private practice, US born, went to US medical schools, talk to patients about CAM, and believe CAM use should be part of routine history. More generalists than sub-specialists talk about CAM with their patients. These results are similar to the study of primary care physicians by Borkan et al [29] where more generalists than sub-specialists referred for CAM. Even though the use of CAM therapies is higher in children with chronic illnesses who see more sub-specialists, the fact that more pediatricians in general practice than sub-specialists talk about CAM may be because, pediatricians in general practice have better communication with their patients, a more open physician-patient relationship, may be more aware of the limitations of conventional medicine and may deal with less severe but more chronic medical problems for which CAM may be more appropriate. Pediatricians' who would refer for CAM are; younger (45 yrs or less), female, use CAM personally and talk to patients about CAM. Female pediatricians have more positive attitudes about CAM than male pediatricians, i.e. use CAM personally, refer for CAM, want more CME courses on CAM and believe CAM should be part of medical school curriculum. This trend continues seven years later and is similar to the study in 1997 of Michigan pediatricians. [26] Studies done by Gordon et al [24] and Levine et al [23] looking at primary care physicians (internist, pediatricians, family physicians and obstetricians-gynecologists) also reported similar findings. One explanation for this may be that females in general (both patients and physicians) seek medical care, especially preventive care, more often than men and have a more holistic approach to health and healing.

Pediatricians are most likely to refer patients for CAM with chronic medical problems such as: headaches, abdominal pain, asthma, and for pain management; when conventional medical therapies failed, behavioral problems (ADHD, nightmares) and psychiatric disorders (depression, anxiety, eating disorders). Similarly, use of CAM is highest among adults and children with chronic medical problems [2-15] thus, indicating that conventional medical therapies alone are often not enough when dealing with chronic medical problems and that use of CAM adjunctively might be an option. Although our study did not specifically ask whether CAM therapies referred for were in conjunction with conventional medical care.

In less than 10% of the time was any one CAM therapy practiced by the respondents and of these the most common ones were: prayer for healing, relaxation (yoga, mediation), massage therapies, imagery, herbs, megavitamins, lifestyle diet and biofeedback. Therapies most often used personally, believed to be most safe, effective and referred for were: acupuncture/acupressure, biofeedback, massage, relaxation (yoga, mediation), hypnosis, chiropractic manipulation, imagery and prayer for healing. Thus pediatricians' use of and attitudes about effectiveness, safety and referral practices were most often consistent. The study by Kemper et al [27] also reported similar findings that the most common professional CAM therapies used personally by pediatricians were: massage and other body works, chiropractic, spiritual/religious healing and acupuncture. Herbs, megavitamins, chiropractic manipulation and homeopathy were considered to be the most harmful. Interestingly, mind-body therapies such as prayer for healing, relaxation techniques (yoga, mediation), imagery, biofeedback and hypnosis are therapies that pediatricians' had a positive attitude about, use personally and would refer for. If one of the goals is to integrate CAM therapies with conventional medical care, then integration of mind-body therapies would be a good start. There are studies indicating the positive effects of mind-body therapies, especially for chronic illness and pain management. [30][31-36,36] Compared to the survey of Michigan pediatricians [26] seven years ago, these respondents to a national survey indicated that they would use/refer and consider safe and effective manipulative therapies such as: acupressure/acupuncture, massage therapies and chiropractic manipulation. This increase in acceptance of these modalities may be as a result of current research showing that they are safe, effective for certain conditions, used by patients and also reimbursed by third party payers [37][38,39].

This study has several limitations. The fact that only one mailing of the survey was done to a national random sample of 3500 pediatricians, with a response rate of 18.5%, raises the possibility of response bias. Despite a fairly good sample size, a second follow up mailing might have been more helpful and produced a higher response rate. Another limitation is that this study only assessed pediatricians' attitudes towards CAM and did not survey nurse practitioners and family physicians who also provide medical care to children. The survey instrument assessed attitudes and referrals in general for CAM but did not ask specifically about referrals/use of CAM therapies for common pediatric problems such as upper respiratory infections, eczema, preventative care, nor did it specify whether referrals for CAM were in conjunction with conventional medical care or alone. Maybe more pediatricians might consider referrals for CAM if they were in conjunction with conventional medical care.

Despite limitations of the study, this study indicates that pediatricians have a positive attitude towards CAM, believe that almost one-third of their patients are using CAM and majority believe there should be more education on CAM, both in medical schools and through CME programs. Even though only a third of pediatricians surveyed ask about CAM use as part of the routine medical history, a majority believe it should a part of the history. Patients are increasingly seeking pediatricians who are knowledgeable about both conventional medicine and CAM. Pediatricians recognize this and want more education in CAM. Thus, medical schools and post-graduate medical training centers have to consider educating and training future pediatricians in the area of CAM. Given the use of CAM by children and the growing evidence of effectiveness of some therapies, some CAM therapies can be integrated into pediatric care. Pediatricians are becoming aware that CAM can not be ignored and that continued education/training and on going research is needed, specifically with regards to children and CAM.

## Authors' contributions

AS was the principle investigator and designed and supervised all aspects of the study including writing and editing the manuscript.

RT performed the statistical analysis, wrote the statistical portion of the manuscript and edited the manuscript. All authors read and approved the final manuscript.

## Pre-publication history

The pre-publication history for this paper can be accessed here:


